# The Prophylaxis and Treatment of Typhoid Fever by the Anti-Typhoid Vaccine

**Published:** 1914-02

**Authors:** 


THE PROPHYLAXIS AND TREATMENT OF TYPHOID FEVER BY THE ANTI-TYPHOID VACCINE. Gregoria Araoz Alfaro, La *Semana Medica,* October 30, 1913, quotes Vincent to the effect that the vaccine causes the formation of a great quantity of antibodies. The agglutinating power oscillates between 1-60 and 1-1000, but may be as high as 1-5000

Block and Creuze studied the blood after the inoculation of the Chantemesse vaccine. At times, 6 days after the first injection, the agglutination reached from 1-100 to 1-500 and more. After a series of injections the agglutinative power increases greatly, even to 1-20,000. In four months it was found to be from 1-200 to 1-1000. After a year in more than half the subjects, it was still 1-100.

In this work Alfaro prefers the Besredka vaccine. He reports a series of typhoid cases—most of them children—to whom this vaccine was administered.

He concludes:

1. That the antityphoid vaccination has demonstrated its efficiency in a manner indisputable and on a large scale. (Especially that of Pfeiffer & Kolle, Russell, Vincent, Wright-Leishman.)

2. He believes that all the American countries should prepare one of these vaccines in sufficient quantities to immunize all who may desire it, and that its use should be compulsory in epidemic or endemic areas and among troops in camp.

3. He believes, further that the method Besredka is destined to produce an immunization more rapid and more lasting than vaccines containing dead organisms.

4. In the treatment of typhoid fever, the Besredka vaccine can be employed with positive results. The reaction produced is negligible. Three to four successive doses of ½ to 1cc in children, and 1, 2 or even 3cc in adults, daily, or with a day's interval, shortens the duration of the illness, improves the general health and hastens the decline of the fever.

It is important to start the treatment promptly and, in the presence of an epidemic or when confronted by a clear picture of the disease, the Widal reaction should not be awaited·

